# Effect of Inhaled Ciclesonide in Non–Critically Ill Hospitalized Patients With Coronavirus Disease 2019: A Multicenter Observational Study in Japan

**DOI:** 10.1093/ofid/ofad571

**Published:** 2023-11-24

**Authors:** Jun Suzuki, Shiro Endo, Takayuki Suzuki, Teppei Sasahara, Shuji Hatakeyama, Yuji Morisawa, Mineji Hayakawa, Kazuma Yamakawa, Akira Endo, Takayuki Ogura, Atsushi Hirayama, Hideo Yasunaga, Takashi Tagami

**Affiliations:** Division of Infectious Diseases, Jichi Medical University Hospital, Shimotsuke, Tochigi, Japan; Division of Infectious Diseases, Tohoku Medical and Pharmaceutical University Hospital, Sendai City, Miyagi, Japan; Department of Infection Prevention and Control, Tohoku Medical and Pharmaceutical University Hospital, Sendai City, Miyagi, Japan; Division of Infectious Diseases, Tohoku Medical and Pharmaceutical University Hospital, Sendai City, Miyagi, Japan; Department of Infection Prevention and Control, Tohoku Medical and Pharmaceutical University Hospital, Sendai City, Miyagi, Japan; Division of Crisis Management Network for Infectious Diseases, Tohoku Medical and Pharmaceutical University, Sendai City, Miyagi, Japan; Division of Infectious Diseases, Jichi Medical University Hospital, Shimotsuke, Tochigi, Japan; Division of Infectious Diseases, Jichi Medical University Hospital, Shimotsuke, Tochigi, Japan; Department of Infection and Immunity, School of Medicine, Jichi Medical University, Shimotsuke, Tochigi, Japan; Division of Infectious Diseases, Jichi Medical University Hospital, Shimotsuke, Tochigi, Japan; Division of General Internal Medicine, Jichi Medical University Hospital, Shimotsuke, Tochigi, Japan; Division of Infectious Diseases, Jichi Medical University Hospital, Shimotsuke, Tochigi, Japan; Department of Emergency Medicine, Hokkaido University Hospital, Kita-ku, Sapporo, Japan; Department of Emergency and Critical Care Medicine, Osaka Medical and Pharmaceutical University, Osaka, Japan; Trauma and Acute Critical Care Center, Tokyo Medical and Dental University Hospital, Bunkyo-ku, Tokyo, Japan; Department of Emergency Medicine and Critical Care Medicine, Tochigi Prefectural Emergency and Critical Care Centre, Imperial Foundation Saiseikai Utsunomiya Hospital, Utsunomiya, Tochigi, Japan; Public Health, Department of Social Medicine, Graduate School of Medicine, Osaka University, Suita, Japan; Department of Clinical Epidemiology and Health Economics, School of Public Health, University of Tokyo, Bunkyo-ku, Tokyo, Japan; Department of Emergency and Critical Care Medicine, Nippon Medical School Musashikosugi Hospital, Kawasaki, Kanagawa, Japan

**Keywords:** COVID-19, SARS-CoV-2, ciclesonide, inhaled corticosteroid, mortality

## Abstract

**Background:**

Coronavirus disease 2019 (COVID-19) is an ongoing global pandemic. Although systemic steroids play an important role in treating patients with severe COVID-19, the role of inhaled corticosteroids in non–critically ill, hospitalized patients with COVID-19 remains unclear.

**Methods:**

We analyzed findings in non–critically ill, hospitalized patients with COVID-19 who were >18 years old and were admitted to 64 Japanese hospitals between January and September 2020. We performed propensity score matching analysis to evaluate 28-day and in-hospital mortality rates with or without inhaled ciclesonide within 2 days of admission. Sensitivity analyses using inverse probability weighting analysis, and generalized estimating equation method were also performed.

**Results:**

Eligible patients (n = 3638) were divided into ciclesonide (n = 290) and control (n = 3, 393) groups. The 1-to-4 propensity score matching analysis included 271 ciclesonide users and 1084 nonusers. There were no significant differences between the 2 groups for 28-day (3.3% vs 2.3%; risk difference, 1.0% [95% confidence interval, −1.2 to 3.3]) or in-hospital (4.8% vs 2.6%; risk difference, 2.2 [−.5 to 4.9]) mortality rates. The sensitivity analysis showed similar outcomes.

**Conclusions:**

From this multicenter observational study in Japan, inhaled ciclesonide did not decrease 28-day or in-hospital mortality rates in non–critically ill, hospitalized patients with COVID-19. Future large, multinational, randomized trials are required to confirm our results.

At the end of 2019, the coronavirus disease 2019 (COVID-19) was identified as the cause of an outbreak of pneumonia in Wuhan, China [1]. COVID-19 remains a global pandemic that often leads to severe acute respiratory syndrome [2, 3]. Strategies for treating COVID-19 have been investigated in many countries to reduce its spread and associated mortality rate [4, 5].

The Randomized Evaluation of COVID-19 Therapy (RECOVERY) trial showed that intravenous dexamethasone was associated with lower mortality in patients hospitalized for COVID-19 [6], and the results of a meta-analysis were similar to those of the RECOVERY trial [7]. Systemic corticosteroids, therefore, are widely used and play an important role in treating patients with severe COVID-19 [4, 5]. However, the strategy of using inhaled corticosteroids against COVID-19 has not yet been established.

Ciclesonide is an inhaled corticosteroid delivered via a nonchlorofluorocarbon hydrofluoroalkane metered-dose inhaler. Ciclesonide is converted into an active metabolite and has beneficial effects in treating asthma [8]. A previous experimental study showed that ciclesonide might have a potent antiviral effect against severe acute respiratory syndrome coronavirus 2 (SARS-CoV-2) [9, 10], and 4 randomized controlled trials (RCTs) have investigated the effects of ciclesonide on COVID-19 [11–14]. Two RCTs focused on outpatients with COVID-19, and 2 focused on hospitalized patients with asymptomatic to moderate COVID-19 [11–14]. However, the latter 2 RCTs on hospitalized patients concluded with inconsistent results among them [12, 14] and there are limited data in the real world clinical data. Thus, the effect of ciclesonide in hospitalized patients remains unclear. The current study aimed to investigate the effects of ciclesonide in non–critically ill patients with COVID-19 in Japan.

## METHODS

The Japanese Multicenter Research on Coronavirus disease 2019 (COVID-19) by Assembling Real-world Data (J-RECOVER study) was a multicenter observational study conducted across 64 Japanese hospitals. The study protocol was reviewed and approved by the ethics committee or investigational review board of each participating center. The need for written informed consent was waived by the ethics committees of all the centers because the data were collected retrospectively. A research outline of the study protocol, including data collection, has been published [15]. This research outline is briefly described in the following sections.

### Data Collection

The current study included patients with SARS-CoV-2 infection diagnosed by means of laboratory testing, who were discharged from each participating hospital between 1 January and 30 September 2020. SARS-CoV-2 was mainly the pre-alpha variant confirmed in Japan during this period [16]. We obtained the following data from case report forms from each participating hospital: age, sex, and race; clinical symptoms, such as dyspnea or hemoptysis; vital signs; pneumonia at admission (defined as confirmed diagnosis using chest radiography or computed tomography); dates of hospital admission, hospital discharge, intensive care unit (ICU) admission, and ICU discharge; COVID-19–associated complications; information about settings or laboratory data before and after initiation of mechanical ventilation, nasal high-flow therapy, or extracorporeal membrane oxygenation; all laboratory data during hospitalization; and center information. After the participating centers prepared a list of cases, the Diagnosis Procedure Combination (DPC) hash application created by the principal investigator of this study was used at each participating center. The application allows the extraction and creation of a DPC data file only for eligible cases from the original DPC data file and anonymizes the data [15].

### DPC Data

The DPC data are administrative claims data obtained from approximately 8 million inpatients discharged per year from >1000 acute care hospitals in Japan. Because the DPC data are linked to a health insurance payment system, the attending physicians accurately recorded the diagnoses, including the main diagnosis and comorbid conditions. The DPC data included the following information on demographics and selected clinical information: admission and discharge, discharge status (live or dead), diagnoses at admission, comorbid conditions at admission, diagnoses after admission, surgical and other procedures performed, dates of surgical or other procedures, medications, dates when medications were administered, and special reimbursements for specific conditions during hospitalization [17].

### Patient Selection

**Figure 1.**
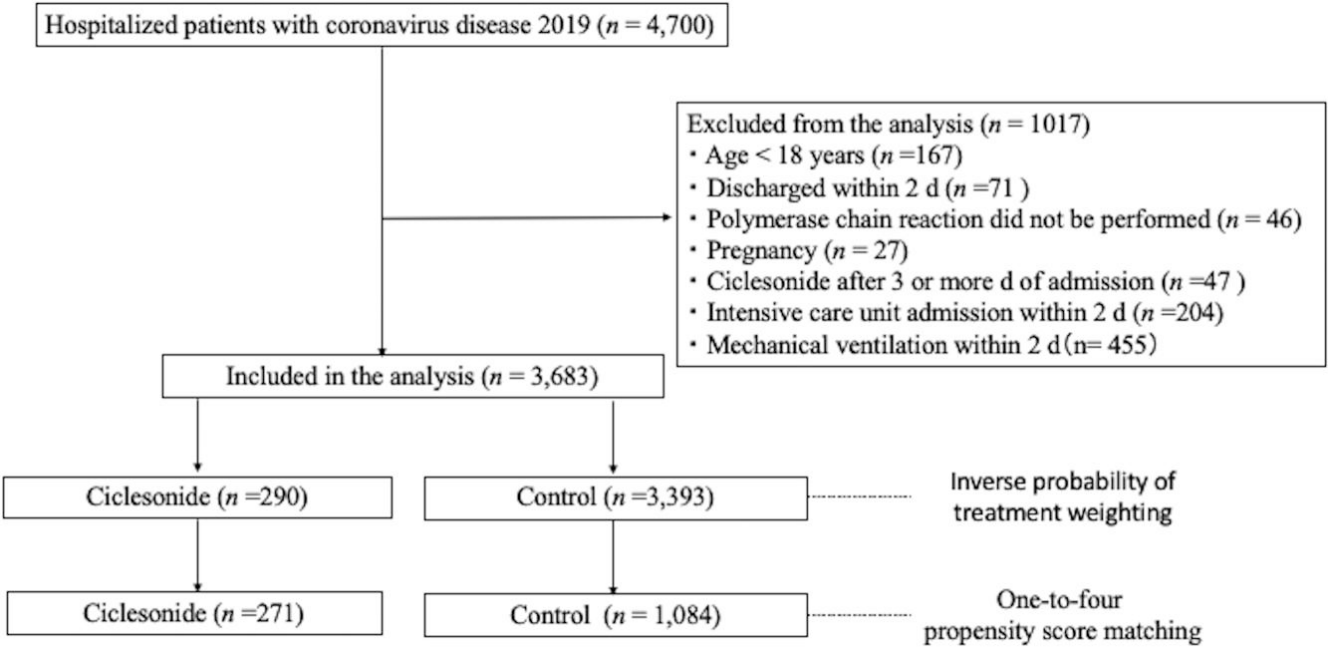
Flow chart representing patient inclusion criteria. Abbreviations: COVID-19, coronavirus disease 2019; ICU, intensive care unit; PCR, polymerase chain reaction.

We identified the non–critically ill patients with COVID-19 during the study periods. Non–critically ill hospitalized patients were defined as hospitalized patients who did not receive mechanical ventilation support or enter the ICU within 2 days after admission, according to the definition of severity in COVID-19 according to the Ministry of Health, Labour and Welfare of Japan [18]. We excluded patients who met the following criteria: age <18 years of age, discharge within 2 days of admission, pregnancy, ciclesonide use from after 3 or more days of admission through discharge, ICU admission within 2 days after hospital admission, or mechanical ventilation within 2 days of admission (Figure 1).

### Variables

The exposure of interest was whether patients received inhaled ciclesonide. The ciclesonide group included patients who received inhaled ciclesonide within 2 days of admission, whereas the control group included patients who did not receive the same. In Japan, physicians prescribe the drugs according to the prescription dose defined by the government. In ciclesonide, the usual dosage and administration for adults is 100–400 μg of ciclesonide once daily by inhalation. Although the ciclesonide dosage can be adjusted according to the patient's symptoms, the maximum daily dose is 800 µg.

The variables assessed included age, age distribution, sex, race, body mass index (calculated as weight in kilograms divided by height in meters squared), hospital type (academic or nonacademic), total number of beds in each hospital, dyspnea, median duration (in days) from symptom onset to admission, pneumonia at admission, and comorbid conditions at admission. Hospital bed information was divided into 3 groups according to interquartile range: <600 beds (interquartile range, <25%), ≥600 and ≦815 beds (≥25% and ≦75%), and 815 beds (75%). Pneumonia at admission was evaluated using chest radiography or computed tomography.

Comorbid conditions at the time of admission were defined according to the Charlson comorbidity index and included myocardial infarction, congestive heart failure, cerebrovascular disease, chronic pulmonary disease, dementia, mild liver disease, malignancy, diabetes mellitus with or without chronic complications, rheumatic diseases, renal disease, and human immunodeficiency virus (HIV) infection. Several elements of the patient medical histories (peripheral vascular disease, peptic ulcer disease, severe liver disease, hemiplegia, and paraplegia) were not included in this analysis because they were relevant to only a few or no patients. We investigated the following severity score and laboratory data; sequential organ failure assessment (SOFA) score, white blood cell count (in cells per microliter), C-reactive protein level (in micrograms per deciliter), and D-dimer level (in micrograms per milliliter). The SOFA score was used to evaluate the severity of COVID-19 at admission. This scoring system is established from 6 organ system categories (hepatic, cardiovascular, respiratory, cardiology, central nervous system, and renal) and is a good predictor of sepsis-related severity [19].

We also investigated the following procedures performed within 2 days of admission: systemic steroids, antiviral therapy, antibiotics, anticoagulation, and oxygen support. Systemic steroid administration included oral and intravenous routes. Antiviral therapy included oral favipiravir and intravenous remdesivir. Antibiotics administered orally and/or intravenously were included. Anticoagulants included heparin, direct oral anticoagulants, and warfarin. Oxygen support included oxygen that was started within 2 days of admission.

### Outcome Measures

The main outcomes of this study were 28-day and in-hospital mortality rates. We also evaluated the following outcomes: proportion of patients transferred to other hospitals, proportion of patients requiring mechanical ventilation from 3 or more days after admission through discharge, and proportion of ICU admissions from 3 or more days after admission through discharge. The proportion of patients transferred to another hospital refers to transfer because the patient's symptoms worsened. In addition, we determined the proportions of deaths due to respiratory events such as worsening pneumonia or the proportions of deaths due to non-respiratory events in in-hospital mortality for each hospital.

### Statistical Analysis

We performed 1-to-4 propensity score matching to adjust for differences in baseline characteristics between the 2 groups. To estimate the propensity score, the probability of a patient receiving ciclesonide was adjusted for potential confounders using the following characteristics: age, age distribution, sex, race, body mass index, hospital information, dyspnea, median duration from symptom onset to admission, pneumonia at admission, comorbid conditions at admission, systemic steroids, antiviral therapy, antibiotics, anticoagulants, and oxygen support within 2 days after admission. The C statistic was calculated to evaluate the goodness of fit. A 1-to-4 matched analysis using nearest-neighbor matching was performed based on the propensity scores. A match occurred when a patient in the ciclesonide group had an estimated score within 0.2 standard deviations of a score for a patient in the control group. We used absolute standardized mean differences to assess the balance between patient characteristics. Absolute standardized mean differences of <10% were considered negligible imbalances in baseline characteristics between the groups [20]. Descriptive data were reported as numbers and percentages for categorical variables and as medians and interquartile ranges for continuous variables. Descriptive statistics were assessed before and after propensity score matching [21].

We also used risk differences to compare outcomes between the groups. In addition, we performed sensitivity analyses using inverse probability of treatment weighting (IPTW) and the generalized estimating equation (GEE) method. We used logistic regression analysis fitted with a GEE-adjusted propensity score to investigate the association between ciclesonide and the outcomes by estimating odds ratios and associated confidence intervals (CIs) using methods appropriate for all included patients [22, 23]. In addition, Kaplan-Meier plots with log-rank statistics were used to assess survival rate differences between patients with or without ciclesonide in the propensity score–matched groups. Multiple imputations were used to correct missing values, using IBM SPSS software, version 28 (IBM SPSS). Propensity score matching, IPTW, GEEs, and Kaplan-Meier analysis were performed using Stata/MP 18.0 software (StataCorp).

## RESULTS

A total of 4291 patients with COVID-19 were identified during the study period. Eligible patients were divided into a ciclesonide group (n = 290) and a control group (n = 3393). The 1-to-4 propensity score matching analysis included the ciclesonide group (n = 271) and the control group (n = 1084). The C statistic indicated that the goodness of fit was 0.709 (95% CI, .68 to .74) in the propensity score model.

Table 1 presents the baseline characteristics of the groups before and after propensity score matching. Patient characteristics were well balanced between the groups after propensity score matching, as the absolute standardized mean differences were <10%. Supplementary Table 1 shows the baseline characteristics after IPTW analysis. Here as well, patient characteristics were well balanced between the groups after IPTW analysis; the absolute standardized mean differences were <10%.

**Table 1.** Patient Characteristics Before and After Propensity Score Matching

Characteristic	Patients in Unmatched Groups, No. (%)^a^		Standardized Difference, %	Patients in Propensity Score–Matched Groups, No. (%)^a^		Standardized Difference, %
	Ciclesonide (n = 290)	Control (n = 3393)		Ciclesonide (n = 271)	Control (n = 1084)	
Age, median (SD), y	48.0 (20.3)	49.0 (19.9)	−4.6	49.0 (20.3)	47.0 (19.6)	3.9
Age group						
<70 y	234 (80.7)	2698 (79.5)	2.7	216 (79.7)	882 (81.4)	−4.2
70–79 y	29 (10.0)	382 (11.3)	−4.0	29 (10.7)	119 (11.0)	−0.9
≥80 y	27 (9.3)	313 (9.2)	0.6	26 (9.6)	83 (7.7)	6.9
Female sex	108 (37.2)	1383 (40.8)	−6.7	104 (38.4)	396 (36.5)	3.8
Japanese ancestry	270 (93.1)	3187 (93.9)	−3.6	252 (93.0)	1018 (93.9)	−3.7
BMI, median (SD)^b^	23.4 (4.5)	23.3 (4.7)	−5.2	23.4 (4.5)	23.1 (4.6)	0.5
Hospital information						
Academic hospital	104 (35.9)	1107 (32.6)	6.4	99 (36.5)	361 (33.3)	6.8
Total no. of beds in hospital						
Median no. (SD)	753 (226)	753 (221)	7.1	753 (225)	756 (215)	0.1
<600 beds	93 (32.1)	1204 (35.5)	−6.9	88 (32.5)	341 (31.5)	2.2
≥600 and ≦815 beds	108 (37.2)	1308 (38.5)	−2.5	102 (37.6)	437 (40.3)	−5.5
815 beds	89 (30.7)	881 (26.0)	9.9	81 (29.9)	306 (28.2)	3.7
Symptoms						
Dyspnea	58 (20.0)	711 (21.0)	−2.2	56 (20.7)	215 (19.8)	2.1
Duration from symptom onset to admission, median (SD), d	5.0 (5.2)	5.0 (5.2)	−4.1	5.0 (5.3)	5.0 (5.2)	−4.8
Pneumonia on admission	166 (57.4)	2170 (64.2)	−3.8	156 (57.6)	640 (59.0)	−3.0
Comorbid conditions						
Myocardial infarction	1 (0.3)	12 (0.4)	0.4	1 (0.4)	3 (0.3)	1.6
Chronic heart failure	2 (0.7)	41 (1.2)	−5.1	2 (0.7)	5 (0.5)	3.6
Cerebrovascular disease	3 (1.0)	36 (1.1)	−0.3	3 (1.1)	7 (0.6)	4.9
Chronic pulmonary disease	15 (5.2)	139 (4.1)	5.1	13 (4.8)	57 (5.3)	−2.1
Dementia	7 (2.4)	58 (1.7)	5.2	6 (2.2)	22 (2.0)	1.3
Mild liver disease	2 (0.7)	45 (1.3)	−6.4	3 (1.9)	13 (2.1)	2.3
Cancer	4 (1.4)	103 (3.0)	−11.3	4 (1.5)	11 (1.0)	4.2
Diabetes mellitus^c^	22 (7.6)	304 (9.0)	−4.9	22 (8.1)	91 (8.4)	−1.0
Rheumatic diseases	2 (0.7)	16 (0.5)	2.9	2 (0.7)	10 (0.9)	−2.0
Renal disease	4 (1.4)	68 (2.0)	−4.9	4 (1.5)	14 (1.3)	1.6
HIV infection	3 (1.0)	32 (0.9)	0.9	3 (1.1)	12 (1.1)	0.0
SOFA score, median (SD)	0.0 (0.6)	0.0 (0.6)	−4.3	0.0 (0.4)	0.0 (0.7)	1.0
Laboratory data						
WBCs, median (SD), 10^9^ cells/L	4.9 (2.7)	5.1 (2.8)	−2.8	4.8 (2.7)	5.0 (2.4)	−0.3
CRP, median (SD), mg/L	1.52 (5.5)	1.78 (5.8)	2.2	1.46 (5.6)	1.63 (6.3)	1.2
D-dimer median (SD), μg/mL	1.19 (5.2)	1.08 (7.6)	2.5	1.20 (5.3)	1.00 (10.7)	−1.3
Treatment within 2 d of admission						
Systemic steroids	64 (22.1)	330 (9.7)	34.6	57 (21.0)	232 (21.4)	−0.9
Antiviral therapy	47 (16.2)	672 (19.8)	−9.2	45 (16.6)	181 (16.7)	−0.2
Antibiotics	126 (43.4)	624 (18.4)	55.7	107 (39.5)	434 (40.0)	−1.1
Anticoagulants	119 (41.0)	562 (16.6)	55.6	102 (37.6)	398 (36.7)	1.9
Oxygen support	26 (9.0)	387 (11.4)	−8.1	25 (9.2)	102 (9.4)	−0.6

Abbreviations: BMI, body mass index; CRP, C-reactive protein; HIV, human immunodeficiency virus; SD, standard deviation, SOFA, sequential organ failure assessment; WBCs, white blood cells.

^a^Data represent no. (%) of patients unless otherwise specified.

^b^BMI calculated as weight in kilograms divided by height in meters squared.

^c^Diabetes mellitus with or without chronic complications.

### Outcome Measures

Table 2 shows the outcomes in the ciclesonide and control groups before and after propensity score matching. Before matching, the 28-day mortality rate was 3.1% in the ciclesonide group versus 2.4% in the control group (risk difference, 0.7% [95% CI, −1.3 to 2.7]), and the in-hospital mortality rate was 4.5% versus 2.9%, respectively (1.6% [−.8 to 4.0]). The proportions of in-hospital deaths due to respiratory events were 92% in the ciclesonide group versus 99% in the control group. The proportions of transfers to other hospital were 1.0% versus 0.27%, respectively (risk difference, 0.8% [95% CI, .9 to 1.5]), the proportions requiring mechanical ventilation after 3 or more days of admission were 0.7% in the versus 0.4 (0.3% [−.4 to 1.1]), and the proportions of ICU admissions after 3 or more days of hospital admission were 3.1% versus 1.8% (1.3% [−.7 to 3.4]).

**Table 2.** Outcome Measures Before and After Propensity Score Matching

Outcome	Ciclesonide Group, No. (%)	Control Group, No. (%)	Risk Difference (95% CI), %
Unmatched group	n = 290	n = 3393	…
28-day mortality	9 (3.1)	82 (2.4)	0.7 (−1.3 to 2.7)
In-hospital mortality	13 (4.5)	99 (2.9)	1.6 (−0.8 to 4.0)
In-hospital deaths due to respiratory events	12/13 (92)	91/99 (91)	…
Transfers to other hospitals	3 (1.0)	9 (0.27)	0.8 (.9 to 1.5)
Mechanical ventilation required after 3 or more d of admission	2 (0.7)	13 (0.4)	0.3 (−.4 to 1.1)
ICU admissions after 3 or more d of admission	9 (3.1)	60 (1.8)	1.3 (−.7 to 3.4)
Propensity score–matched group	n = 271	n = 1084	…
28-day mortality	9 (3.3)	25 (2.3)	1.0 (−1.2 to 3.3)
In-hospital mortality	13 (4.8)	28 (2.6)	2.2 (−.5 to 4.9)
In-hospital deaths due to respiratory events	12/13 (92)	27/28 (96)	…
Transfers to other hospitals	3 (1.1)	3 (0.28)	0.8 (−.5 to 1.7)
Mechanical ventilation required after 3 or more d of admission	2 (0.7)	5 (0.4)	0.3 (−.8 to 1.3)
ICU admissions after 3 or more d of admission	9 (3.3)	16 (1.5)	1.8 (−.4 to 4.1)

Abbreviation: CI, confidence interval; ICU, intensive care unit.

After propensity score matching, there were no significant differences between the 2 groups for 28-day mortality rate (3.3% for ciclesonide vs 2.3% for control group; risk difference, 1.0% [95% CI, −1.2 to 3.3]), in-hospital mortality rate (4.8% vs 2.6%, respectively; risk difference, 2.2 [−.5 to 4.9]), proportion of transfers to other hospitals (1.1% vs 0.28%; risk difference, 0.8% [−.5 to 1.7]), the proportion requiring mechanical ventilation after 3 or more days of admission (0.7% vs 0.4%, risk difference, 0.3% [−.8 to 1.3]), or the proportion of ICU admissions after 3 or more days of admission (3.3% vs 1.5%; risk difference, 1.8% [−.4 to 4.1]). The proportions of in-hospital deaths due to respiratory events were 92% in the ciclesonide group versus 96% in the control group.

**Figure 2.**
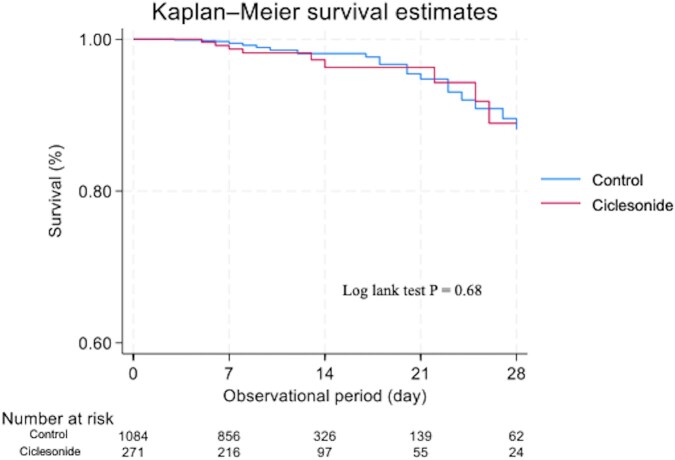
Kaplan-Meier survival plots for patients treated with or without ciclesonide within 2 days of admission in propensity score–matched groups. No significant difference in survival rate was observed between ciclesonide and control groups (log-rank χ^2^, 0.16; *P* = .68).

The risk differences in IPTW analysis were similar after propensity score matching (Supplementary Table 2). The Kaplan-Meier survival plots after admission for the differences between the ciclesonide and control groups (propensity score–matched groups) are shown in Figure 2. There were no significant difference between the 2 groups (log-rank χ^2^ = 0.16; *P* = .68).

Table 3 shows the association between ciclesonide use and outcomes using the generalized estimating equation (GEE) analysis appropriate for all included patients. GEEs showed that there were no significant differences between the 2 groups for the 28-day mortality rate (odds ratio, 1.4 [95% CI, .68–2.8)], the in-hospital mortality rate (1.8 [.97–3.3), the proportion of transfers to other hospital (3.1 [.8–13), the proportion requiring mechanical ventilation after 3 or more days of admission (1.4 [.30–6.9), or the proportion of ICU admissions after 3 or more days of admission (1.8 [.86–3.8]).

**Table 3.** Association of Ciclesonide With Outcomes Based on Generalized Estimating Equations Analysis Adjusted for Propensity Score in All Included Patients

Outcome	OR (95% CI)
28-d Mortality rate	1.4 (.7– 2.8)
In-hospital mortality rate	1.8 (.97–3.3)
Proportion of transfers to other hospitals	3.1 (.8–13)
Proportion requiring mechanical ventilation after 3 or more d of admission	1.4 (.3–6.9)
Proportion of ICU admissions after 3 or more d of admission	1.8 (.9–3.8)

Abbreviations: CI, confidence interval; ICU, intensive care unit; OR, odds ratio.

## DISCUSSION

In this multicenter observational study involving 4291 hospitalized, non–critically ill patients with COVID-19, inhaled ciclesonide did not significantly decrease the 28-day mortality rate, the in-hospital mortality rate, the proportion of transfers to another hospital, the proportion of patients requiring mechanical ventilation after 3 or more days of admission, or the proportion of ICU admissions after 3 or more days of admission.

There are multiple possible reasons why inhaled ciclesonide failed to improve mortality rates. First, it may be associated with a high risk of bacterial pneumonia exacerbation in patients with COVID-19. In several studies, inhaled steroid use has been shown to increase the risk of bacterial colonization and bacterial pneumonia [24, 25]. A previous systematic review and meta-analysis showed that inhaled corticosteroids may increase the risk of pneumonia in patients with chronic lung disease [24]. In addition, an earlier RCT in patients with mild COVID-19 showed a risk ratio of worsened pneumonia of 2.08 (95% CI, 1.15–3.75) in the ciclesonide group [14]. In our study, although data on pneumonia exacerbation could not be investigated, the ICU admission rate was higher in the ciclesonide group. In addition, almost all in-hospital deaths were due to respiratory events (Table 2). Therefore, ciclesonide may be associated with worsening COVID-19, canceling its the beneficial effect.

Second, inhaled ciclesonide may have low systemic bioavailability and may not have adequate antiviral or anti-inflammatory effects to improve the prognosis in patients with COVID-19. Although the antiviral mechanism of ciclesonide against SARS-CoV-2 remains unknown, some previous studies have shown that ciclesonide inhibits viral endoribonuclease, activated kinase, or the viral RNA replication-transcription complex, which might suppress viral replication [26]. In addition to its antiviral effects, ciclesonide has potential anti-inflammatory effects against lung injury, preventing progression to severe pneumonia and acute respiratory distress syndrome [9]. Inhaled ciclesonide can be delivered to lung tissues at high concentrations; however, it is not absorbed into the bloodstream. Therefore, ciclesonide has low systemic bioavailability, leading to a lower potential effect [8]. Thus, antiviral and anti-inflamammary effects may not be adequate against COVID-19, and ciclesonide may not be associated with recovery.

Third, different inhaled corticosteroids may differ in their effectiveness. Two RCTs have investigated the effects of budesonide as an inhaled glucocorticoid in patients with COVID-19. The PRINCIPLE trial showed that budesonide was associated with improved time to recovery, lower rates of hospital admission, and fewer deaths [27]. The STOIC trial also showed that budesonide reduced the time to self-reported recovery and the rate of emergency room visits [28]. In contrast, in the ACTIV-2 study, inhaled fluticasone did not show a shorter recovery time compared with the control group among outpatients with COVID-19 [29]. Our study showed that inhaled ciclesonide was not associated with improving mortality rates. Therefore, the differences in the mechanisms of action of budesonide and ciclesonide may be responsible for this difference in effects and requires further investigation.

Fourth, the timing of ciclesonide initiation may be associated with these results. A previous study showed that initiation of ciclesonide within 3 days after diagnosis was associated with prevention of mechanical ventilation [12], and inhaled corticosteroids might be most effective in the early phase of COVID-19. However, in our study, the duration from symptom onset to admission was approximately 5 days in the propensity score­–matched groups. Therefore, the later timing of ciclesonide initiation may be associated with these results.

Our study has several strengths. First, our results are reflective of a real-world clinical setting and treatment strategies for COVID-19 in Japan. Second, many participating hospitals were tertiary emergency centers with an average number of beds >750 (Table 1), and they could provide a higher level of care. Third, compared with previous studies investigating the effects of ciclesonide, our study included the largest number of non–critically ill patients with COVID-19.

Our study also has several limitations. First, it was a retrospective multicenter study, and confounding indications may have influenced the results. Second, our database did not include any information on long-term symptoms associated with COVID-19, and we did not know the effect of ciclesonide against long COVID syndrome [30]. Third, our participating centers were mainly tertiary emergency centers; because patients with mild illness and a lower mortality risk could not easily be admitted to these hospitals, our results may not be generalizable to other hospitals. Fourth, this study was not based in the SARS-CoV-2 vaccine era, and there may be different results in that era [31]. Fifth, our database lacked information about ciclesonide use before admission, which may have influenced our results. Sixth, we used systemic steroid administration within 2 days of admission in our analysis. We did not exclude systemic steroids from our analysis because a previous retrospective study showed that systemic steroids were not associated with in-hospital mortality rates in non–severely ill hospitalized patients with COVID-19 [32]. However, the use of this factor in analysis may have influenced our results. Seventh, data were not available on the number of beds for patients with COVID-19 in each hospital. Finally, although propensity score matching ensured an adequate balance in patient characteristics between the groups, confounding factors due to unmeasured covariates cannot be completely avoided; hence, bias cannot be ruled out.

In conclusion, in this multicenter observational study in Japan, inhaled ciclesonide did not reduce 28-day or in-hospital mortality rates in non–critically ill hospitalized patients with COVID-19. Future large, multinational, randomized trials are required to confirm our results.

## Supplementary Data

Supplementary materials are available at *Open Forum Infectious Diseases* online. Consisting of data provided by the authors to benefit the reader, the posted materials are not copyedited and are the sole responsibility of the authors, so questions or comments should be addressed to the corresponding author.

## Supplementary Material

ofad571_Supplementary_Data
