# Supplementary material for: Effect of Inhaled Ciclesonide in Non–Critically Ill Hospitalized Patients With Coronavirus Disease 2019: A Multicenter Observational Study in Japan
Source: Open Forum Infect Dis. 2023 Nov 24;10(12):ofad571. doi: 10.1093/ofid/ofad571 (PMC10709541; doi:10.1093/ofid/ofad571)
Supplement: ofad571_Supplementary_Data [file ofad571_supplementary_data.zip › Supplemental Table 1.docx]

**Supplemental Table 1. Patient characteristic in the IPTW analysis group.**

|  | IPTW analysis group | |  |
| --- | --- | --- | --- |
|  | Ciclesonide  group | Control  group | Standardized difference (%) |
|  | *n* = 3,542 | *n* = 3,570 |  |
| **Characteristic** |  |  |  |
| Age (year), median（SD) | 50.0 (20.0) | 49.0 (20.0) | 2.8 |
| Distribution, n (%) |  |  |  |
| <70 years | 2823 (79.6) | 2922 (81.8) | 0.2 |
| 70-79 years | 375 (10.6) | 410 (11.5) | -1.9 |
| >=80 years | 344 (9.7) | 338 (9.5) | 1.7 |
| Sex (Female), n (%) | 1391 (39.2) | 1481 (41.5) | -2.2 |
| Race (Japanese), n (%) | 3375 (95.2) | 3446 (96.5) | 5.6 |
| Body mass index (kg/m^2^), median (SD) | 23.6 (4.5) | 23.2 (4.7) | -1.7 |
| **Hospital information** |  |  |  |
| Academic hospital, n (%) | 1183 (33.4) | 1204 (33.7) | 1.2 |
| Total number of beds in each hospital (beds) median (SD). | 747 (226) | 753 (221) | 0.2 |
| < 600 beds, n (%) | 1224 (34.6) | 1291 (36.1) | -1.3 |
| 600 =< and < 815 beds, n (%) | 1314 (37.1) | 1411 (39.5) | -2.8 |
| 815 beds =<, n (%) | 1004 (28.3) | 967 (27.1) | 4.4 |
| **Symptoms** |  |  |  |
| Dyspnea, n (%) | 668 (18.9) | 765 (21.4) | -4.9 |
| Median number of days from symptom onset to admission, day (SD) | 5.0 (5.6) | 5.0 (5.2) | -1.6 |
| Pneumonia on admission, n (%) | 2206 (62.3) | 2336 (65.4) | -2.8 |
| **Comorbidities, n (%)** |  |  |  |
| Myocardial infarction | 15 (0.4) | 12 (0.3) | 1.8 |
| Chronic heart failure | 21 (0.6) | 42 (1.2) | -5.6 |
| Cerebrovascular disease | 55 (1.5) | 39 (1.1) | 4.9 |
| Chronic pulmonary disease | 166 (4.7) | 154 (4.1) | 2.2 |
| Dementia | 56 (1.6) | 64 (1.7) | -1.0 |
| Mild liver disease | 71 (2.0) | 47 (1.3) | 7.2 |
| Malignancy | 67 (1.9) | 107 (3.0) | -6.9 |
| Diabetes with or without chronic complications | 325 (9.2) | 325 (9.1) | 1.1 |
| Rheumatic diseases | 23 (0.6) | 18 (0.5) | 2.0 |
| Renal disease | 71 (2.0) | 72 (2.0) | 0.3 |
| HIV infection | 36 (1.0) | 35 (1.0) | 0.6 |
| **Severity score** |  |  |  |
| SOFA score, median (SD) | 0.0 (0.6) | 0.0 (0.7) | -0.3 |
| **Laboratory data** |  |  |  |
| White blood cells (/μL), median (SD) | 4700 (2493) | 5080 (2827) | -7.6 |
| C-Reactive protein (mg/L), median (SD) | 1.8 (5.2) | 1.76 (5.8) | -1.2 |
| D-Dimer (μg/mL), median (SD) | 1.2 (5.0) | 1.0 (7.7) | 2.5 |
| **Treatment within 2 days of admission, n (%)** |  |  |  |
| Systemic steroids | 495 (14.0) | 394 (11.0) | 9.0 |
| Antiviral therapy | 721 (20.4) | 717 (20.1) | 2.1 |
| Antibiotics | 796 (22.5) | 750 (21.0) | 4.5 |
| Anticoagulants | 731 (20.6) | 680 (19.0) | 4.8 |
| Oxygen support | 412 (11.6) | 414 (11.6) | 1.2 |

*Abbreviations*: HIV, human immunodeficiency virus: IPTW, inverse probability of treatment weighting SD, standard deviation, SOFA: sequential organ failure assessment.
