# Supplementary material for: Effect of Inhaled Ciclesonide in Non–Critically Ill Hospitalized Patients With Coronavirus Disease 2019: A Multicenter Observational Study in Japan
Source: Open Forum Infect Dis. 2023 Nov 24;10(12):ofad571. doi: 10.1093/ofid/ofad571 (PMC10709541; doi:10.1093/ofid/ofad571)
Supplement: ofad571_Supplementary_Data [file ofad571_supplementary_data.zip › Supplemental Table 2.docx]

**Supplemental Table 2. The outcome measures in IPTW analysis.**

|  | IPTW analysis group |
| --- | --- |
|  |  |
|  |  |
| **Outcomes, n (%)** | Risk difference  (95% Confidence intervals) |
| 28-day mortality | 0.8% (-1.4 to 3.0) |
| In-hospital mortality | 1.7% (-0.9 to 4.5) |
| Proportion of transfers to other hospitals | 0.4% (-0.4 to 8.1) |
| Proportion of requirement of mechanical ventilation after 3 days of admission | -0.16% (-0.5 to 0.2) |
| Proportion of ICU admissions after 3 days of admission | 1.5% (-1.1 to 4.3) |

*Abbreviations*: Confidence interval; ICU, intensive care unit; IPTW, inverse probability of treatment weighting.
